# Biocontrol Efficacy of *Bacillus velezensis* FXJ Against *Fusarium graminearum*-Induced Fusarium Head Blight in Wheat

**DOI:** 10.3390/jof12010037

**Published:** 2026-01-02

**Authors:** Yihua Liao, Xiao Xu, Huijuan Peng, Ao Chen, Chenjingzi Hao, Chengcheng Li

**Affiliations:** 1 MARA Key Laboratory of Sustainable Crop Production in the Middle Reaches of the Yangtze River (Co-Construction by Ministry and Province)/Hubei Key Laboratory of Waterlogging Disaster and Agricultural Use of Wetland, College of Agriculture, Yangtze University, Jingzhou 434025, China; liaoyihua0815@163.com (Y.L.); 2024720966@yangtzeu.edu.cn (H.P.); chenao19991007@163.com (A.C.); 2025720946@yangtzeu.edu.cn (C.H.); 2 Jiangsu Academy of Agricultural Sciences, Jiangsu Coastal Area Institute of Agricultural Sciences, Yancheng 224000, China; ycxx202509@163.com

**Keywords:** *Fusarium graminearum*, *Bacillus velezensis* FXJ, antifungal activity, deoxynivalenol, Fusarium head blight management

## Abstract

Fusarium head blight (FHB), caused by *Fusarium graminearum*, poses a serious threat to wheat production and grain security. In this study, a strain of *Bacillus velezensis* was isolated from the plant *Polygonatum sibiricum* and designated FXJ. FXJ inhibited the mycelial growth of *F. graminearum* by 52% and induced hyphal abnormalities including swelling and shrinkage. *In vivo* experiments demonstrated that FXJ treatment significantly reduced disease severity in wheat coleoptiles and spikes, decreased deoxynivalenol accumulation in grains, and down-regulated the expression. Transcriptomic analysis further revealed that FXJ suppressed fungal growth by interfering with energy metabolism and essential biosynthetic processes, particularly pathways related to fatty acid degradation and sugar metabolism. Overall, *B. velezensis* FXJ shows strong potential for integrated management of wheat Fusarium head blight through combined mechanisms, including the inhibition of mycelial growth, disruption of hyphal morphology, reduction in pathogen infection, and suppression of toxin synthesis.

## 1. Introduction

Wheat (*Triticum aestivum* L.) is one of the most important food crops worldwide. However, changes in climate and cultivation practices have contributed to the large-scale emergence and spread of Fusarium head blight (FHB), a devastating wheat disease that poses a serious threat to global wheat production and food safety [1]. FHB is primarily caused by the *Fusarium graminearum* species complex (FGSC), with *F. graminearum* and *F. culmorum* being the most significant pathogens [2]. Infection can occur at all growth stages, leading to seed rot, seedling rot, stem rot, and ear rot, with ear rot causing the greatest damage. The disease causes substantial yield losses of up to 60%. FHB contaminates wheat with mycotoxins such as deoxynivalenol (DON), which present serious risks to food safety and animal health [2]. In recent years, both the incidence and severity of FHB have increased globally, and the International Maize and Wheat Improvement Center (CIMMYT) has recognized FHB as a major factor affecting grain quality and yield [3].

The limited availability of naturally FHB-resistant germplasm has rendered conventional breeding methods insufficient for developing resistant wheat varieties [4]. Currently. Wheat resistance against *Fusarium* spp. is generally classified into two main types: resistance to penetration and resistance to spread after infection. Penetration resistance relies on preformed structural and biochemical barriers, such as cell wall thickening and reinforcement through deposits of callose and lignin, which physically block initial fungal invasion [5]. In contrast, resistance to spread is an induced defense response that involves the synthesis and accumulation of phytoalexins (e.g., deoxynivalenol), activation of pathogenesis-related proteins, and other biochemical processes to restrict further pathogen colonization within host tissues [6]. Notably, the specific resistance background of the host plant can significantly influence the interaction efficiency with biocontrol agents, thereby critically affecting the overall efficacy of biological control [7]. FHB management relies largely on chemical pesticides, with biological control serving as a complementary strategy. However, chemical control has significant drawbacks, including a singular mode of action, environmental pollution and ecological degradation from long-term overuse, and the emergence of pathogen resistance. For instance, field resistance to carbendazim and phenamacril has exacerbated the severity and expanded the range of FHB [8,9,10,11]. Consequently, the identification of highly effective and environmentally sustainable biocontrol agents has become a critical strategy for managing FHB. Among these, *Bacillus velezensis* has emerged as a particularly promising candidate due to its multifaceted abilities to suppress plant pathogens, promote plant growth, and enhance crop stress tolerance [12].

*B. velezensis* is an aerobic, Gram-positive, endospore-forming bacterium widely recognized as a plant growth-promoting rhizobacterium (PGPR) [13]. This bacterium exhibits strong antibacterial activity, effectively inhibiting a broad range of plant pathogenic microorganisms. Moreover, it can induce systemic acquired resistance (SAR) in plants, thereby enhancing host defense responses. *B. velezensis* also secretes various growth-promoting compounds, including phytohormones and siderophores, which directly support plant growth and development [14]. Its ability to synthesize diverse functional enzymes and bioactive secondary metabolites further expands its applications in enzyme production, food fermentation, environmental bioremediation, and bioenergy development [15]. As an efficient and environmentally sustainable alternative for phytopathogen control, microbial biocontrol agents have attracted increasing attention. In recent years, *B. velezensis* has emerged as a leading biocontrol strain and has been widely adopted in multiple countries [16]. Notably, the active spores of *B. velezensis* strain FZB42 have been commercially formulated as the bioinoculant RhizoVital^®^, which has shown remarkable efficacy in managing soil-borne diseases [17].

In this study, we investigated the biocontrol potential of a *B. velezensis* strain FXJ, isolated from the rhizosphere soil of *Polygonatum sibiricum*, against FHB caused by *F. graminearum*. The objectives of this study were to: (1) identify the species of the *Bacillus* strains; (2) evaluate its *in vitro* antifungal activity and effects on DON production by *F. graminearum*; and (3) elucidate its biocontrol mechanisms through transcriptomic analysis.

## 2. Materials and Methods

### 2.1. Strains and Culture Conditions

The wild-type *F. graminearum* strains PH-1, PH-2, and PH-3, along with the strain FXJ isolated from the rhizosphere soil of *P. sibiricum* by our research group, were maintained at the Molecular Biology Laboratory, College of Agriculture, Yangtze University. FXJ was cultured in Luria–Bertani (LB) medium containing 10 g/L tryptone, 5 g/L yeast extract, 10 g/L sodium chloride, and 15 g/L agar. *F. graminearum* was grown on potato dextrose agar (PDA) medium consisting of 200 g/L potato, 18 g/L glucose, and 15 g/L agar.

### 2.2. In Vitro Antagonism Assay

The antagonistic activity of strain FXJ against *F. graminearum* was evaluated using the dual-culture method [18]. Mycelial plugs (5 mm) of *F. graminearum* strains PH-1, PH-2, and PH-3 were placed individually at the center of fresh PDA plates (90 mm). Two symmetrical wells were punched at 2.5 cm from the center, and each well was loaded with 40 µL of FXJ suspension (OD_600_ = 0.6, 1 × 10^8^ CFU/mL). Control plates received sterile medium. All treatments were performed in triplicate. The plates were incubated at 25 °C for 4 d. Once the mycelium of the pathogenic fungus in the control group had fully covered the plate, the colony diameter of the pathogen in each treatment was measured, and the inhibition rate was calculated as follows: Inhibition rate (%) = [(Colony diameter of control group − Colony diameter of treatment group)/Colony diameter of control group] × 100.

### 2.3. Identification of Strain FXJ

FXJ was streaked onto LB agar and incubated overnight at 37 °C for observation of colony morphology [19]. Molecular identification was performed by sequencing the *16S rRNA* gene (amplified with primers 27F/1492R) and the *gyrB* gene (amplified with UP1f/UP2r) [20]. PCR products were verified by gel electrophoresis and sequenced by Sangon Biotech (Shanghai, China). Sequences were compared using BLAST (https://blast.ncbi.nlm.nih.gov/Blast.cgi) on NCBI, and a phylogenetic tree was constructed using the Maximum Likelihood method in MEGA 11.0 with 1000 bootstrap replicates.

### 2.4. Effect of Bacterial Cell-Free Supernatant on the Mycelial Growth

Cell-free supernatant (CFS) was prepared as previously described [21]. FXJ was cultured in LB broth at 37 °C and 200 rpm for 12 h, and the OD_600_ was adjusted to 0.8. One milliliter of the preculture was transferred to 100 mL of fresh LB and incubated with shaking for 4 d. The fermentation broth was centrifuged at 12,000 rpm for 15 min, and the supernatant was filter-sterilized (0.22 µm) to obtain CFS. CFS was incorporated into PDA at final concentrations of 1%, 5%, 10%, and 15% (*v*/*v*), with PDA without CFS serving as the control. Plates were incubated at 25 °C for 4 d. Colony diameter and inhibition rate were measured as described in Section 2.2.

### 2.5. Morphological Characteristics of F. graminearum Mycelium

Hyphal morphology was examined using scanning electron microscopy (SEM) (VEGA3, Tescan, Brno, Czech Republic). Hyphal blocks from the edge of the inhibition zone were fixed in 2.5% glutaraldehyde for 20 min, rinsed with distilled water, dehydrated through a graded ethanol series (50%, 70%, 80%, 90%, and 100%), treated with tert-butanol, freeze-dried, sputter-coated with gold, and observed under SEM.

### 2.6. Control Efficacy of FXJ Against FHB in Wheat

Seeds of wheat cultivar Yangmai 158 were surface-sterilized with 2% NaOCl for 10 min, rinsed, germinated on moist filter paper, and transferred to trays. Seedlings with coleoptiles 2–3 cm long were selected, and roots were trimmed. A total of 10 µL of FXJ suspension (OD_600_ = 0.6) was applied to the coleoptile. After 24 h, 10 µL of *F. graminearum* spore suspension (1 × 10^6^ CFU/mL) was inoculated at the same site. Plants were maintained at 25 °C for 6–7 d, and lesion length was measured.

For spike inoculation, FXJ suspension (OD_600_ = 0.6, 1 × 10^8^ CFU/mL) was sprayed onto spikes at the flowering stage. After 24 h, 10 µL of spore suspension was injected into the fifth spikelet from the base. Spikes were misted, bagged for 2 d, and disease severity was assessed at 14 d post-inoculation. Grains were collected for DON analysis.

### 2.7. Determination of DON Content in Wheat Grains

Grain samples were ground under liquid nitrogen and extracted with acetonitrile/water (84: 16, *v*/*v*). The mixture was vortexed for 30 min, sonicated for 1 min, and vortexed again for 30 min. Extracts were passed through a solid-phase extraction column, and 800 µL of the eluate was vacuum-dried. The residue was derivatized with a trimethylsilyl reagent, mixed with isooctane and water, and the supernatant was filtered prior to analysis by GC/MS. DON content was calculated using the following formula: C = (C_0_ × 1.8)/m, where C is the DON concentration (µg/g), C_0_ is the concentration determined by GC/MS, and m is the sample weight (g).

### 2.8. RNA Extraction and Gene Expression Analysis

Total RNA was isolated under strictly maintained conditions at 4 °C. Briefly, 0.5 g of sample was pulverized in liquid nitrogen and homogenized with TRIZOL reagent. Following homogenization, phase separation was induced using pre-chilled chloroform, and the RNA was precipitated with an equal volume of pre-chilled isopropanol. The resulting RNA pellet was subsequently washed with pre-chilled 75% ethanol, air-dried, and finally dissolved in DEPC-treated water for storage at −80 °C. The quality and quantity of RNA were assessed by agarose gel electrophoresis and NanoDrop (Thermo Fisher Scientific, Waltham, MA, USA), respectively. First-strand cDNA was synthesized from 1 μg of RNA using the HiScript IV RT SuperMix for qPCR kit (Vazyme Biotech Co., Ltd., Nanjing, China). Gene expression levels were calculated using the 2^−ΔΔCT^ method, with the actin gene serving as the reference control, as reported previously [22,23]. Each RT-PCR assay was performed in triplicate with three biological replicates. Primer sequences are listed in Table S1.

### 2.9. RNA-Seq Analysis and Validation

*F. graminearum* was cultured in mung bean broth for 3 d at 25 °C and 200 rpm. Spores (10^6^ CFU/mL) were co-cultured with FXJ (OD_600_ = 0.6, 1 × 10^8^ CFU/mL) in YEPD broth for 48 h. Total RNA was extracted using the RNAprep Pure Plant Kit (Tiangen, Beijing, China), and RNA integrity was evaluated with the RNA Nano 6000 Assay Kit on an Agilent Bioanalyzer 2100 system (Agilent Technologies, Santa Clara, CA, USA). Qualified RNA samples were subsequently sequenced on the Illumina NovaSeq platform.

Raw sequencing data in FASTQ format (raw reads) were preprocessed using an in-house Perl script to obtain high-quality clean reads. Clean reads were aligned to the reference genome using Hisat. Differentially expressed genes (DEGs) were identified using DESeq2 with thresholds of |log_2_(fold change)| ≥ 2, *p* ≤ 0.01, and false discovery rate (FDR) < 0.01 [24]. Functional annotation and enrichment analysis of DEGs were performed using the Gene Ontology (GO; http://www.geneontology.org (accessed on 16 September 2025)) and Kyoto Encyclopedia of Genes and Genomes (KEGG; https://www.genome.jp/kegg (accessed on 16 September 2025) databases. To validate the sequencing results, ten DEGs were selected and experimentally verified by qRT-PCR using the 2^−ΔΔCT^ method with gene-specific primers (Supplementary Table S1).

### 2.10. Statistical Analysis

Statistical analyses were performed using SPSS Statistics software (version 20; IBM Corp., Armonk, NY, USA). Comparisons between groups were conducted using Tukey’s honestly significant difference test [25]. The bar heights represent the mean values of biological replicates, and the error bars indicate the standard error of the mean.

## 3. Results

### 3.1. Antifungal Activity of FXJ Against F. graminearum

**Figure 1:**
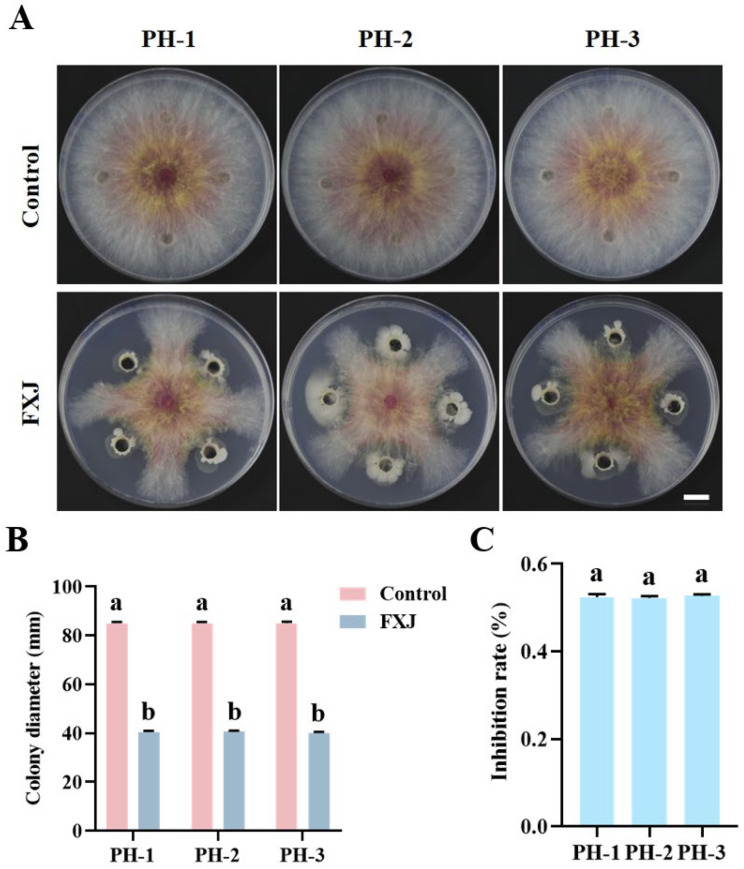
Antifungal activity of strain FXJ against *Fusarium graminearum*. (**A**) Mycelial growth of *Fusarium graminearum* under the FXJ treatment. Colony diameter (mm) (**B**) and Inhibition rate (**C**) of strain FXJ against *Fusarium graminearum*. Scale bar: 1 cm. All experiments were repeated three times with consistent results. Tukey’s honestly significant difference analysis of variance was used for statistical analysis. Different letters above the bars indicate significant differences (*p* < 0.05).

Strain FXJ exhibited strong inhibitory activity against *F. graminearum* (including strains PH-1, PH-2, and PH-3). Dual-culture assays showed that FXJ inhibited mycelial growth by 52% across all tested strains, with no significant differences among groups (Figure 1).

### 3.2. Identification of Strain FXJ

**Figure 2:**
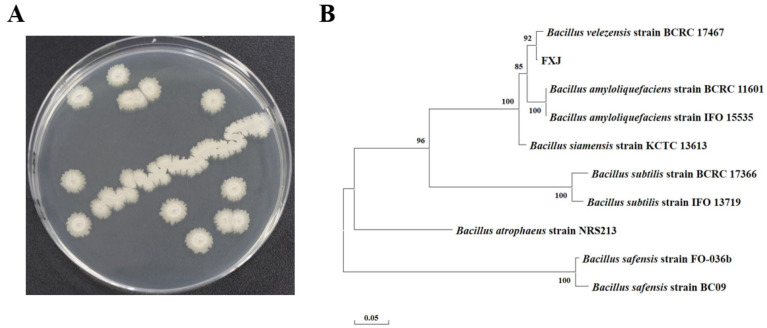
Morphology and phylogenetic tree of strain FXJ. (**A**) The colonial morphology of strain FXJ in LB medium. (**B**) Phylogenetic tree of FXJ based on the 16S *rRNA* and *gyrB* sequence and closely related species constructed by the Maximum likelihood method. The initial tree was automatically generated by applying the Neighbor-Joining and BioNJ algorithms to a matrix of pairwise distances estimated using the Tamura–Nei model, followed by the selection of the topology with the superior log-likelihood value. The number at the nodes indicates the level of bootstrap support (%) based on 1000 replicates. The scale bar at the bottom indicates the genetic distance.

After 24 h of growth on solid LB medium, strain FXJ formed opaque, milky-white colonies with rough, wrinkled surfaces and irregular margins (Figure 2A). To determine its taxonomic identity, molecular identification was conducted. The sequenced *16S rRNA* and *gyrB* genes were analyzed using BLAST, and sequences from 10 closely related Bacillus strains were selected as references for phylogenetic tree construction. The results showed that FXJ clustered with *B. velezensis* with a bootstrap support value of 92 (Figure 2B). Based on colony morphology and molecular phylogenetic analysis, strain FXJ was identified as *B. velezensis*.

### 3.3. Suppression of F. graminearum Mycelial Growth Suppression by CFS

**Figure 3:**
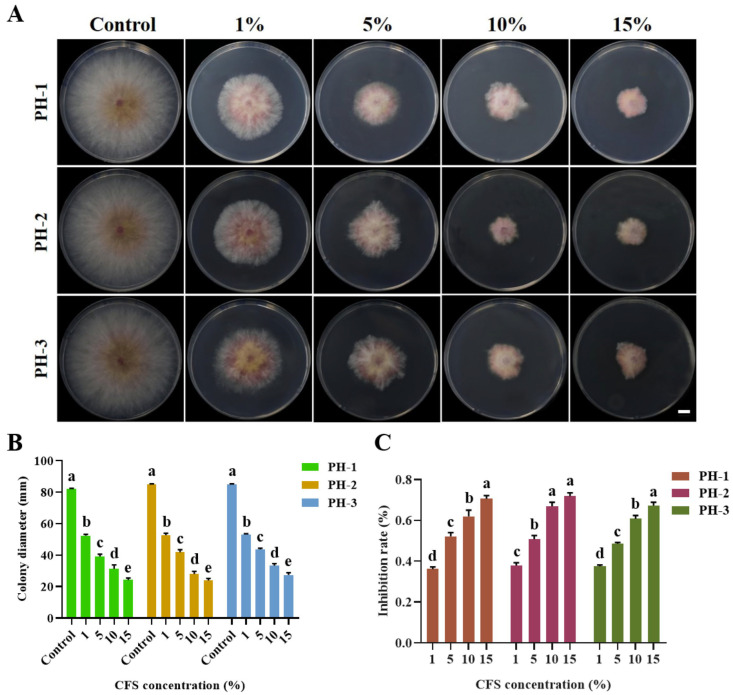
Antifungal effect of strain FXJ on *Fusarium graminearum* determined using different concentrations of CFS *in vitro*. (**A**) Mycelial growth of *Fusarium graminearum* on PDA under CFS treatment. Colony diameter (mm) (**B**) and mycelial inhibition rate (**C**) of *Fusarium graminearum* under CFS treatment. Scale bar: 1 cm. All experiments were repeated three times with consistent results. Tukey’s honestly significant difference analysis of variance was used for statistical analysis. Different letters above the bars indicate significant differences (*p* < 0.05).

The CFS of FXJ significantly inhibited the mycelial growth of *F. graminearum* (Figure 3). Antifungal activity increased with higher CFS concentrations. When the CFS concentrations reached 16%, the inhibition rate against *F. graminearum* reached 70% (Figure 3C).

### 3.4. Effects of FXJ on F. graminearum Mycelial Morphology

**Figure 4:**
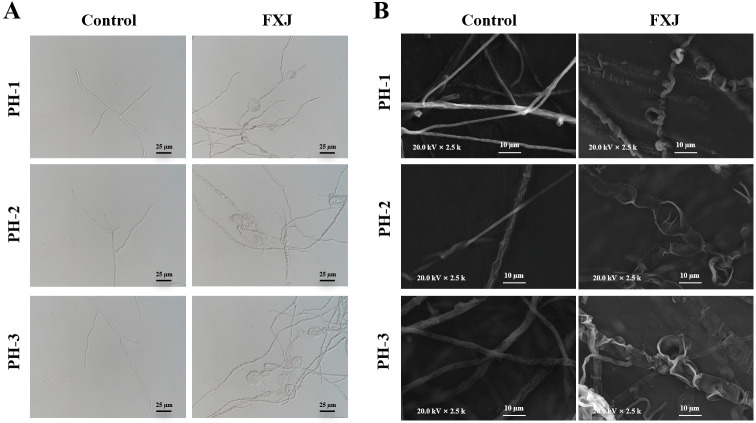
The effect of FXJ treatment on the mycelial morphology of *Fusarium graminearum*. The control group was treated with sterile water. (**A**) Optical microscopy and scanning electron microscopy (**B**).

Morphological changes in untreated and FXJ-treated *F. graminearum* mycelia were evaluated using optical microscopy and SEM. The mycelia in the control group exhibited normal morphology, uniform thickness, and orderly distribution. FXJ treatment induced pronounced swelling in the middle and terminal regions of some hyphae, accompanied by collapse and shrinkage of the mycelial structures (Figure 4).

### 3.5. Control Efficacy of Strain FXJ Against Wheat FHB

**Figure 5:**
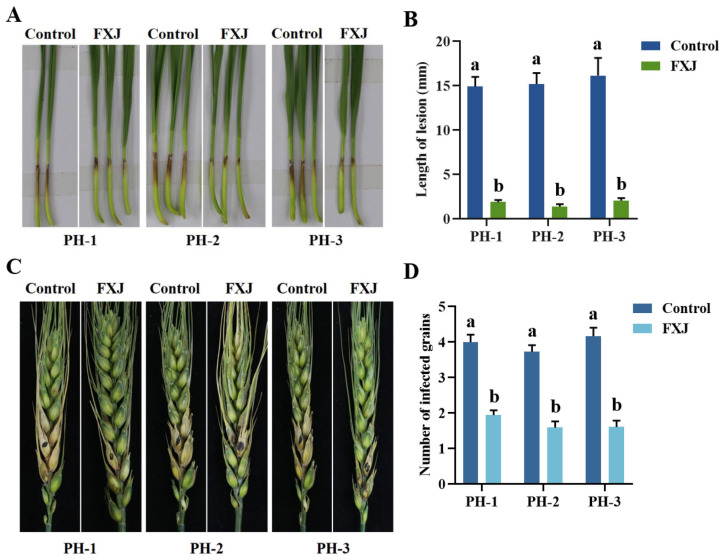
Control efficacy of strain FXJ against wheat Fusarium head blight. (**A**) Representative images of coleoptiles captured 5 d after infection with *Fusarium graminearum*. (**B**) Statistical analysis of lesion length on wheat coleoptiles infected by *Fusarium graminearum*. (**C**) Representative images of wheat spikes captured 14 d after infection with *Fusarium graminearum*. (**D**) Statistical analysis of the number of diseased grains in infected spikes. All experiments were repeated three times with consistent results. Tukey’s honestly significant difference analysis of variance was used for statistical analysis. Different letters above the bars indicate significant differences (*p* < 0.05).

The effect of FXJ on the infectivity of *F. graminearum* was evaluated using wheat coleoptile and single-floret inoculation assays (Figure 5). FXJ treatment significantly reduced pathogen infectivity in both coleoptiles and wheat spikes. In the control group, the lesion length at the base of coleoptiles reached approximately 15 mm, with four diseased grains observed on infected spikes. In contrast, the FXJ-treated group exhibited a lesion length of only about 2 mm and fewer diseased grains, with only two grains affected.

### 3.6. Effects of FXJ on DON Toxin Synthesis

**Figure 6:**
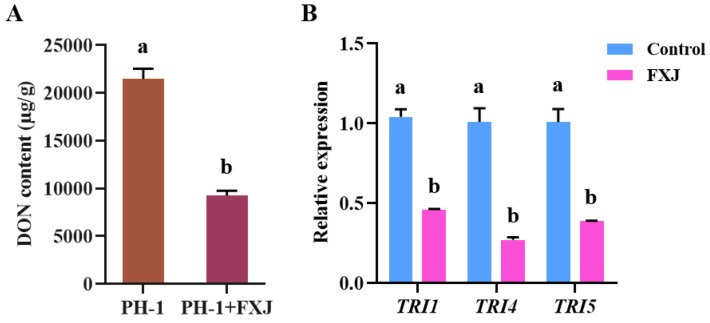
Effects of strain FXJ on DON biosynthesis and expression of TRI genes in *Fusarium graminearum* PH-1. (**A**) DON production in infected wheat grains. (**B**) Expression levels of *TRI1*, *TRI4*, and *TRI5* genes in *Fusarium graminearum* PH-1 under co-culture with strain FXJ or alone. Data are presented as the mean ± standard deviation (SD) from three independent experiments (N = 3). Tukey’s honestly significant difference analysis of variance was used for statistical analysis. Different letters above the bars indicate significant differences (*p* < 0.05).

DON is a key virulence factor that facilitates fungal spread within wheat heads [26]. To determine whether FXJ influences DON biosynthesis in *F. graminearum* PH-1, DON content in infected wheat grains treated with FXJ was compared with untreated controls. FXJ treatment significantly reduced DON accumulation in the infected grains compared to the control group (Figure 6A). Furthermore, qRT-PCR analysis revealed that FXJ treatment markedly down-regulated the expression of key DON biosynthetic genes, including *TRI1*, *TRI4*, and *TRI5* (Figure 6B).

### 3.7. Transcriptomics and Functional Enrichment of FXJ-Treated F. graminearum

**Figure 7:**
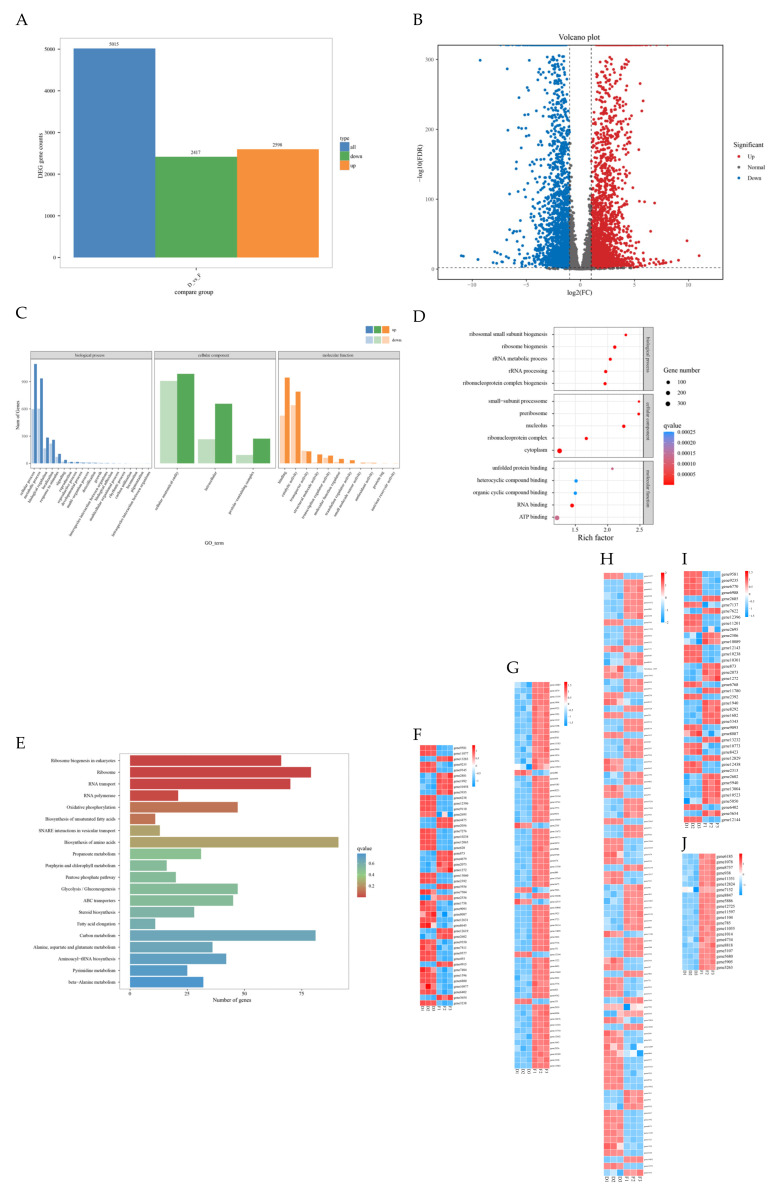
Overview of gene expression changes in *Fusarium graminearum* PH-1 after treatment with strain FXJ. (**A**) Bar chart showing the number of up- and down-regulated differentially expressed genes (DEGs) in pairwise comparisons. (**B**) Volcano plot of DEGs between the control and FXJ-treated groups. Red dots represent up-regulated DEGs, blue dots represent down-regulated DEGs, and gray dots indicate non-DEGs. GO functional classification, KEGG classification of DEGs for Control vs. FXJ and Heatmap of the KEGG pathway. (**C**) Histogram of GO functional classification. The DEGs were assigned to three categories: biological process, cellular component, and molecular function. (**D**) GO functional classification map of the first five terms with small q value in biological process, molecular function, and cellular component. The color of the points reflects the *p* value, and the red indicates the more significant enrichment. The size of the dots represents the count of DEGs enriched. (**E**) Histogram of KEGG classification. (**F**) Heatmap of DEGs in glycolysis/gluconeogenesis pathway (ko00010). (**G**) Heatmap of DEGs in fatty acid degradation pathway (ko03008). (**H**) Heatmap of DEGs in amino acid biosynthesis pathway (ko01230). (**I**) Heatmap of DEGs in ribosome biogenesis in eukaryotes pathway (ko00071). (**J**) Heatmap of DEGs in RNA polymerase pathway (ko03020).

DEGs between the control and treatment groups are shown. Using the thresholds of |log_2_(fold change)| ≥ 2 and FDR ≤ 0.01, a total of 5015 DEGs were identified, including 2417 down-regulated and 2598 up-regulated genes (Figure 7A,B).

GO functional classification comprises three main categories: biological process, molecular function, and cellular component. GO enrichment analysis revealed that the largest number of DEGs was annotated under biological processes, covering the broadest range. Specifically, 1692 and 1538 DEGs were associated with cellular processes and metabolic processes, respectively. Within the cellular component category, DEGs were significantly enriched in cellular anatomical structures, intracellular components, and protein-containing complexes. In the molecular function category, 1470 and 1431 DEGs were related to binding and catalytic activity, respectively (Figure 7C).

To further explore the potential functions of the DEGs, enrichment analysis was performed on the top five terms with the lowest q-values in the biological process, molecular function, and cellular component categories. In the biological process category, DEGs were significantly enriched in ribosome biogenesis (4.96%), rRNA processing (4.57%), ribonucleoprotein complex biogenesis (4.44%), rRNA metabolic process (3.33%), and ribosomal small subunit biogenesis (1.96%). Within the cellular component category, DEGs were significantly enriched in the cytoplasm (16.70%), nucleus (17.48%), ribonucleoprotein complex (3.42%), preribosome (1.71%), and small-subunit processome (1.04%). In the molecular function category, DEGs were mainly enriched in ATP binding (12.11%) (Figure 7D).

KEGG enrichment analysis was performed to identify the top 20 pathways with the lowest q-values (Figure 7E). A total of 47 genes were mapped to the glycolysis/gluconeogenesis pathway (ko00010), where *GAPDH*, *TPI*, *ALDO*, *HKs*, *GPI*, and *ADH1* were significantly down-regulated, while *DLD*, *ACS*, *HMGSH*, *PEPS*, and *GCK* were significantly up-regulated (Figure 7F). The fatty acid degradation pathway (ko03008) was enriched with 41 genes (Figure 7G). Additionally, 91 genes were enriched in the amino acid biosynthesis pathway (ko01230) (Figure 7H). The most significantly enriched pathway was eukaryotic ribosome biogenesis (ko00071), which accounted for 4.18% of the annotated genes. This pathway included *RIO2*, *eIF6*, *NOP5*, *CDF5*, *BMS1*, and 54 other up-regulated genes (Figure 7I). Moreover, all 19 genes in the RNA polymerase pathway (ko03020) were up-regulated in the FXJ treatment group, including six RNAP subunits (Figure 7J).

Integrated GO and KEGG enrichment analyses indicated that the products of many DEGs were significantly enriched in the “Genetic Information Processing” and “Metabolism” pathway categories, suggesting that strain FXJ may inhibit the growth of *F. graminearum* primarily by modulating nutrient metabolism and genetic information processing.

### 3.8. qRT-PCR Validation of Transcriptome Data

**Figure 8:**
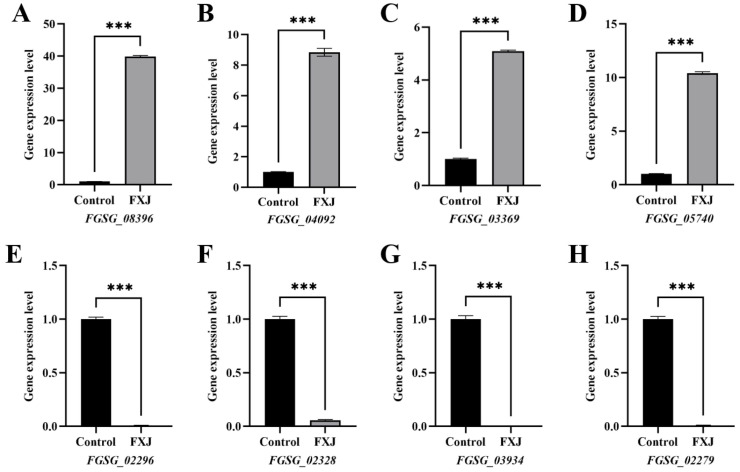
Validation of differentially expressed genes (DEGs) from transcriptome sequencing by RT-qPCR. (**A**) *FGSG_08396*. (**B**) *FGSG_04092*. (**C**) *FGSG_03369*. (**D**) *FGSG_05740*. (**E**) *FGSG_02296*. (**F**) *FGSG_02328*. (**G**) *FGSG_03934*. (**H**) *FGSG_02279*. Asterisks indicate significant differences at *p* < 0.001 (***).

The expression patterns of eight DEGs (four up-regulated and four down-regulated) were highly consistent between RNA-seq and qRT-PCR results, confirming the reliability of the transcriptome data (Figure 8).

## 4. Discussion

FHB primarily caused by *F. graminearum*, is a globally prevalent fungal disease. It reduces wheat yield and contaminates grains with mycotoxins, posing serious threats to food security and human health [27,28]. Owing to its safety, low toxicity, and reduced risk of inducing pathogen resistance, biological control has attracted considerable attention and is increasingly applied in FHB management [29,30,31]. In this study, *B. velezensis* FXJ exhibited strong antagonistic activity against *F. graminearum*. These findings not only expand the microbial resources available for the biological control of FHB but also highlight FXJ as a promising candidate strain for eco-friendly management of the disease.

*B. velezensis* exhibits broad-spectrum antifungal activity and is non-pathogenic to plants [32]. In this study, a strain designated FXJ was isolated from *P. sibiricum* and demonstrated significant inhibitory effects against *F. graminearum*. Morphological characteristics and phylogenetic analysis identified FXJ as *B. velezensis*. Antifungal assays indicated that both the fermentation broth and CFS of FXJ effectively suppressed the mycelial growth of *F. graminearum*, with the CFS achieving up to 70% inhibition, suggesting that the antagonistic activity is primarily mediated by secreted antimicrobial metabolites. It is well established that Bacillus species suppress plant pathogens through the production of lipopeptide antibiotics, which are key determinants of their biocontrol efficacy. For instance, *B. velezensis* JCK-7158 displays broad antagonistic activity against diverse plant pathogenic fungi, mainly due to the production of iturin A and surfactin [33]. Similarly, genomic analysis of *B. velezensis* YB-130 reveals multiple genes encoding hydrolases and secondary metabolites predicted to play important roles in antagonizing pathogenic fungi [34].

FXJ treatment also induced significant morphological abnormalities in the hyphae of *F. graminearum*, including swelling, collapse, and shrinkage in both the middle and apical regions. These changes suggest that FXJ may disrupt cell wall or membrane integrity or interfere with regulatory pathways governing hyphal growth. Such structural damage is commonly associated with the activity of antimicrobial peptides or cell wall-degrading enzymes, aligning with the typical antifungal mechanisms reported in biocontrol strains of the genus *Bacillus* [35].

In *in vivo* inoculation assays, FXJ treatment significantly reduced the infection capacity of *F. graminearum* on wheat coleoptiles and spikes, as indicated by shorter lesion lengths and fewer diseased grains, highlighting its potential for practical FHB control. Importantly, FXJ treatment also markedly decreased DON accumulation in infected grains and down-regulated the expression of key biosynthesis genes (*TRI1*, *TRI4*, and *TRI5*). Since DON is a critical virulence factor that promotes fungal spread within host tissues and poses risks to human and animal health [36]. its suppression is a major target in the biological control of FHB. The strong efficacy of FXJ in inhibiting DON biosynthesis is therefore particularly noteworthy. Although biocontrol agents exhibit remarkable antifungal and mycotoxin-degrading potential under laboratory conditions, their field efficacy is significantly constrained by environmental variables. The application potential of these strains stems from their multiple disease-suppression mechanisms, such as producing antimicrobial lipopeptides, competing for ecological niches, and inducing systemic resistance, which theoretically render them more adaptable than strains with a single mode of action [37]. However, their practical limitations are equally notable. Firstly, temperature and humidity are critical determinants of successful colonization. Suboptimal conditions, such as low temperatures or persistent rainfall during the wheat susceptibility period, can severely inhibit the proliferation and activity of biocontrol agents at key infection sites [38]. Secondly, the complex indigenous microflora in field soils poses a major challenge. Resident microbes compete intensely with introduced biocontrol agents for nutrients and space, and if the latter fail to rapidly establish a dominant population, their efficacy will be substantially compromised [39].

FXJ treatment significantly activated the ribosome biogenesis and amino acid biosynthesis pathways, suggesting an overactivation of the protein synthesis system that may lead to excessive consumption of intracellular resources [40]. At the same time, the down-regulation of key genes in the glycolysis/gluconeogenesis pathway indicates impaired energy production, which cannot meet the elevated demands of biosynthetic processes [41]. This contradiction between hyperactive biosynthesis and insufficient energy supply, combined with the increased transcriptional burden due to up-regulation of the RNA polymerase pathway, drives the pathogen into a state of energy and resource exhaustion, thereby inhibiting its growth and pathogenicity.

In summary, *B. velezensis* FXJ inhibits the growth and pathogenicity of *F. graminearum* through multiple mechanisms, including direct suppression of mycelial expansion, disruption of hyphal morphology, reduction in infectivity, and inhibition of toxin synthesis. These attributes highlight FXJ as a highly promising biocontrol strain for FHB management. Due to certain experimental limitations, the primary role of these antimicrobial substances in disease suppression will be determined based on the systematic isolation and identification of the active metabolites produced by the biocontrol strain FXJ in future studies, and evaluate its efficacy under field conditions to support practical applications.

## Data Availability

The raw sequence data reported in this paper have been deposited in the Genome Sequence Archive (Genomics, Proteomics & Bioinformatics 2025) in National Genomics Data Center (Nucleic Acids Res 2025), China National Center for Bioinformation/Beijing Institute of Genomics, Chinese Academy of Science (GSA: CRA030665) and are publicly accessible at https://ngdc.cncb.ac.cn/gsa (accessed on 12 November 2025).

## Supplementary Materials

The following supporting information can be downloaded at: https://www.mdpi.com/article/10.3390/jof12010037/s1, Table S1: Primers used in the study.
