# Supplementary material for: Biocontrol Efficacy of Bacillus velezensis FXJ Against Fusarium graminearum-Induced Fusarium Head Blight in Wheat
Source: J Fungi (Basel). 2026 Jan 2;12(1):37. doi: 10.3390/jof12010037 (PMC12842974; doi:10.3390/jof12010037)
Supplement: Supplementary file 1 [file jof-12-00037-s001.zip › jof-3932262-supplementary.pdf]

Supporting Information

Table S1. Primers used in the study.

Primer	Sequence (5'-3')	Application
27F	AGAGTTTGATCMTGGCTCAG	Amplification of *16S rRNA* (NR_116240.1)
1492R	GGTTACCTTGTTACGACT	
UP1f	GAAGTCATCATGACCGTTCTGCA	Amplification of *gyrB* (DQ903176.1)
UP2r	AGCAGGGTACGGATGTGC	
FGSG_08396-F	CGTATTATTGCTCCAGGCTTCA	Quantitative real-time PCR of *FGSG_08396* expression (XM_011322078.1, Amino sugar and nucleotide sugar metabolism)
FGSG_08396-R	GGATGTTTTTGCCGTATTCG	
FGSG_04092-F	AATCCTGCGGAAAGTCAAGAG	Quantitative real-time PCR of *FGSG_04092* expression
FGSG_04092-R	CGGTAACTGTAGGTGAAGCCAT	(XM_011323246.1, Steroid biosynthesis)
FGSG_03369-F	CCCGCATTCCAGAAACACT	Quantitative real-time PCR of *FGSG_03369* expression
FGSG_03369-R	AAAGCACCACATTGACCAGC	(XM_011324060.1)
FGSG_05740-F	ACGGATGGTCCCAGAGTTTT	Quantitative real-time PCR of *FGSG_05740* expression
FGSG_05740-R	GGTTTGATGCCGATGTTGTG	(XM_011326020.1, Steroid biosynthesis)
FGSG_02296-F	GGCGTCTGTGCTGCTATTGT	Quantitative real-time PCR of *FGSG_02296* expression
FGSG_02296-R	TGCTGAAAGGGCTGAACCA	(XM_011319903.1, Glycolysis/Gluconeogenesis)
FGSG_02328-F	CTGGTGGATTGGGAGGATAAG	Quantitative real-time PCR of *FGSG_02328* expression
FGSG_02328-R	AGGCATCGGACGGTCTTTT	(XM_011319935.1, Ascorbate and aldarate metabolism)
FGSG_03934-F	GCCTCAATCATAGGAAGCACC	Quantitative real-time PCR of *FGSG_03934* expression
FGSG_03934-R	CCATAGTAATCGCAAGCCCAC	(XM_011323431.1, Meiosis)
FGSG_02279-F	AGCAAGTTGTCAGTTCCGAGC	Quantitative real-time PCR of *FGSG_02279* expression
FGSG_02279-R	AGGGTGTCCGTTTTGAGTGTC	(XM_011319884.1, Glycine, serine and threonine metabolism)
TRI1-F	ACCAGGTCAAGAATGCTCGC	Quantitative real-time PCR of *TRI1* expression (XNO29053.1)
TRI1-R	GGACAGATACGGCAGAAAGGA	
TRI4-F	TATTGTTGGCTACCCCAAGG	Quantitative real-time PCR of *TRI4* expression (AAM49052.1)
TRI4-R	GCCTTGAGAACCTTGACTCG	
TRI5-F	GAGTGTTTCATGCATGGCTACGTC	Quantitative real-time PCR of *TRI5* expression (AAN05032.1)
TRI5-R	CTGAGCCTCCTTCACATCGTCC	
FgActin-F	AACATTGTCATGTCTGGTGGTACC	Internal parameters of quantitative real-time PCR
FgActin-R	CACTTGCGGTGAACGATTGA	(XP_011327086.1)
